# Voice Activity Detection in Noisy Environments Based on Double-Combined Fourier Transform and Line Fitting

**DOI:** 10.1155/2014/146040

**Published:** 2014-08-06

**Authors:** Jinsoo Park, Wooil Kim, David K. Han, Hanseok Ko

**Affiliations:** ^1^Department of Biomicrosystem Technology, Korea University, Anam-dong, Seongbuk-gu, Seoul 136-713, Republic of Korea; ^2^School of Computer Science Engineering, Incheon National University, Songdo-dong, Yeonsu-gu, Incheon 406-772, Republic of Korea; ^3^Office of Naval Research, Arlington, VA 22203, USA; ^4^School of Electrical Engineering, Korea University, Anam-dong, Seongbuk-gu, Seoul 136-713, Republic of Korea

## Abstract

A new voice activity detector for noisy environments is proposed. In conventional algorithms, the endpoint of speech is found by applying an edge detection filter that finds the abrupt changing point in a feature domain. However, since the frame energy feature is unstable in noisy environments, it is difficult to accurately find the endpoint of speech. Therefore, a novel feature extraction algorithm based on the double-combined Fourier transform and envelope line fitting is proposed. It is combined with an edge detection filter for effective detection of endpoints. Effectiveness of the proposed algorithm is evaluated and compared to other VAD algorithms using two different databases, which are AURORA 2.0 database and SITEC database. Experimental results show that the proposed algorithm performs well under a variety of noisy conditions.

## 1. Introduction

The purpose of voice activity detection (VAD) or speech endpoint detection is to determine the beginning and ending points of a speech signal. VAD is an important step in the signal flow of speech recognition. Accurate VAD can not only improve the accuracy of speech recognition but also reduce the complexity of calculation [1, 2]. VAD has been studied for decades, and many algorithms have been proposed, such as hidden Markov models [3], infor(mation entropy [4], wavelet transform technology [5], and variations of these algorithms.

The signal*-*to*-*noise ratio (SNR) was usually taken as an energy cue for discrimination [6, 7]. Besides these simple ones, some advanced energy*-*based features, such as Teager energy [8] and long*-*term speech information [9], were derived by enhancing the discrimination between speech and nonspeech. Developing a VAD for noisy environments with low SNR or for any nonstationary noise is still very challenging. Therefore, abundant VAD algorithms have been developed to achieve better performance in real noise environments. Recently, many VAD methods focus on statistical models to discriminate speech and nonspeech. Most statistical models aimed to construct classifiers for speech and nonspeech classification. The conventional classifier*-*based method employs the Gaussian statistical model with the discrete Fourier transform (DFT) analysis [10, 11]. Based on these researches, multiple observations [12] and multiple statistical models [13] were utilized to further improve the classifiers' performance, respectively. Furthermore, contextual information derived from multiple observations has been incorporated into the likelihood ratio test (LRT) to improve the robustness of VAD under adverse acoustic environment [14]. A novel VAD based on a multivariate complex Gaussian observation model along with definition of an optimal LRT had been presented [15]. Besides the classical methods, a few statistical models aimed at finding the change points between speech and nonspeech. Speech detection can also be accomplished by localizing a rapidly changing edge point over the features of activity and inactivity zones. Accordingly, Canny's edge detector, which was previously utilized to detect the edges of an image, is applied to detect speech [16]. For this purpose, an algorithm based on edge detection filter and state transition model is applied to the frame energy normalization feature for speech endpoint detection [17]. In addition, Gaussian mixture models have been applied to model the static harmonicstructure information and the long*-*term temporal information of speech. VAD decisions are then based on the log likelihood ratios computed from the speech and noise Gaussian mixture model (GMM) [18]. However, these VAD algorithms suffer high computational complexity for two specific reasons. Firstly, they assume that speech and noise are distributed by the Gaussian distribution in the DFT domain. Secondly, noise estimation and adaptation algorithms are considered to improve its robustness under nonstationary noise environments at the cost of additional computation. Therefore, in this paper, a new feature set which is robust for noisy speech environment while keeping low*-*complexity for real*-*time implementation is proposed. The new feature based VAD is further made stable in performance by applying an edge detection filter. In particular, a combination of double-combined Fourier transform (DCFT) and subsequent envelope line fitting is proposed as a feature set such that the pattern of the feature envelope between speech and nonspeech regions becomes more distinguishable, yielding more stable detection results. Experimental evaluations confirm the potential performance of the proposed algorithm under various and real noise environments. The structure of this paper is as follows. In Section 2, the conventional algorithms of VAD are reviewed.  Section 3 describes the proposed VAD algorithm based on DCFT and feature envelope line fitting. In Section 4, the performance evaluation is made through representative experiments. Lastly, Section 5 presents the conclusions of this paper.

## 2. Review of Conventional Feature Models for VAD

In this section, we review the conventional VAD approaches proposed by [17, 18], which are based on frame energy normalization and GMM mapping based on static harmonic feature. The main contribution of our approach will be described in Section 3.

### 2.1. Frame Energy Feature Based Edge Detection Filter with State Transition Model

In endpoint detection using a simple edge detection filter, the objective is to find the edges, which are abruptly changing the feature points caused by existence of speech. It is used to find the edge component with large change of frame energy with the notion that energy increases in speech beginning region and decreases in speech ending region. In addition, final speech endpoint detection is done by applying the results of edge detection filter to state transition model [17].

**Figure 1:**
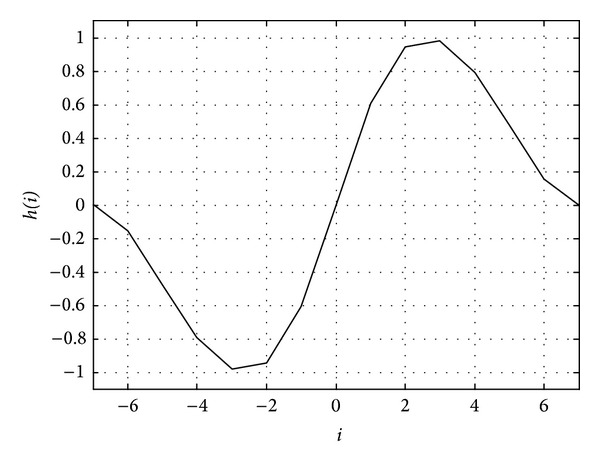
Edge detection filter* h *(*W* = 7).

As shown in Figure 1, since an edge filter is an odd function, if the value of the energy feature in nonspeech region varies smoothly and constantly regardless of its magnitude, the filter output approximates it to zero. It is also notable that if the energy feature value increases at the beginning of speech region, the filter output increases. On the contrary, if the energy feature value decreases at the ending point of speech region, the subsequent filter output also decreases.

The edge detection filter *h* can be defined as in

(1) h(i)={−f(i),−W≤i≤0f(i),1≤i≤W,f(x)=eAx(Z1sin⁡Ax+Z2cos⁡⁡x) +e−Ax(Z3sin⁡Ax+Z4cos⁡⁡x)+Z5+Z6esx,

where *W* represents the length of the filter, *i* is an integer between −*W* and* W*, and* A* and *Z* are filter parameters, respectively. For *W* = 7 and the filter parameters provided by [11] as* A = *0.41, [*Z*_1_, *Z*_2_, *Z*_3_, *Z*_4_, *Z*_5_, *Z*_6_] = [1.538, 1.468, −0.078, −0.036, −0.872, −0.56]. Figure 1 is an example of the filter response when *W* = 7.

The edge detection filter output *F*(*n*) can be calculated by applying the frame energy *g*(*n*) to the edge detection filter *h* as in

(2) $$\begin{array}{c} F\left(n\right)=\overset{W}{\underset{i=-W}{\sum }}h\left(i\right)g\left(n+i\right), \end{array}$$

where *n* is the frame number.

Final beginning and ending points decision needs to be made by comparing the value of *F*(*n*) with some predetermined thresholds. Due to the sequential nature of the detector and the complexity of the decision procedure, we use a state transition model to make the final decisions.

**Figure 2:**
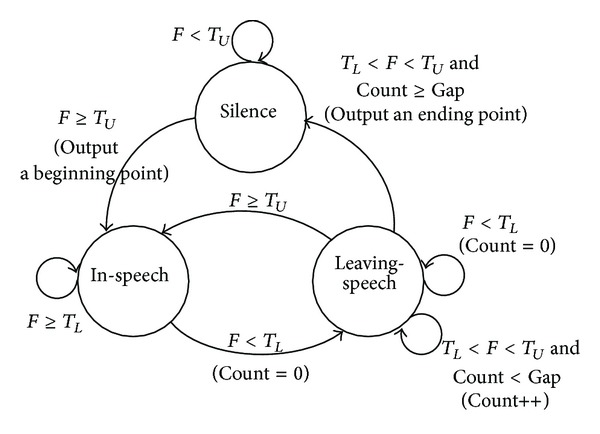
State transition model diagram for endpoint detection [17].

**Figure 3:**
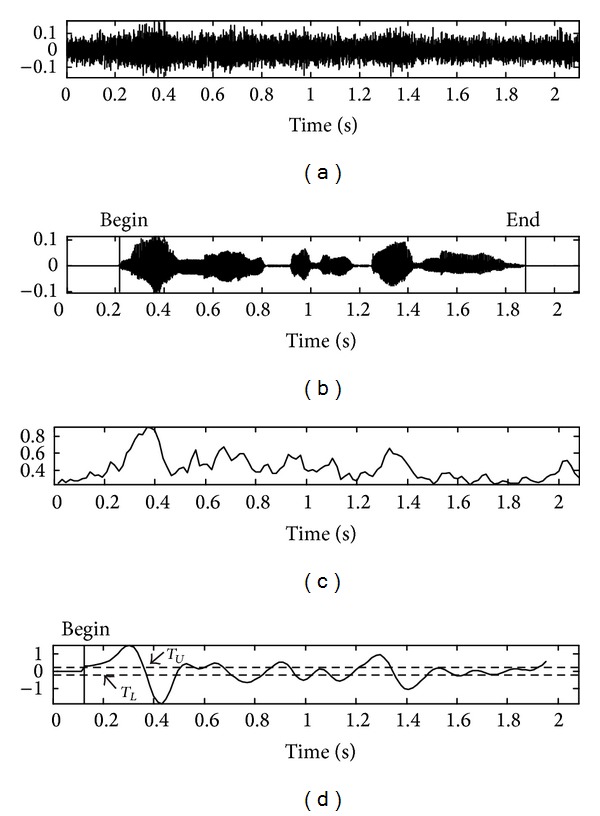
Results of endpoint detection based on frame energy and employing an edge detection filter in a car*-*noise environment: (a) noisy speech signal at SNR −5 dB, (b) clean speech signal, (c) frame energy of signal (a), and (d) detection output of edge filter with state transition model (*T*_*L*_ is lower threshold; *T*_*U*_ is upper threshold).

Figure 2 shows the state transition model to detect the beginning and ending points of a speech signal. As shown in Figure 2, the three states indicated include silence, in*-*speech, and leaving*-*speech. Silence and in*-*speech represent the nonspeech and speech region, respectively. Leaving*-*speech is a state belonging to speech region but has the possibility of turning into a nonspeech region. Inthe state transition model, the input is *F*(*n*) and the output is the detected frame numbers of beginning and ending points. Count is a frame counter, *T*_*L*_ (lower threshold) and *T*_*U*_ (upper threshold) are two thresholds with *T*_*U*_ > *T*_*L*_, and Gap is an integer indicating the required number of frames from a detected endpoint to the actual ending of speech. By assuming that silence state is the starting state, the state diagram stays in the silence state until *F*(*n*) < *T*_*U*_. For cases of *F*(*n*) ≥ *T*_*U*_, it can be said that a beginning point is detected and the state goes into the in*-*speech state. During the stay in the in*-*speech state, it moves to the leaving*-*speech state and sets Count = 0 if *F*(*n*) < *T*_*L*_. It stays in the leaving*-*speech state where Count < Gap and then moves to the silence state where Count ≥ Gap. At silence state, it can be said that an ending point is detected. It is noted that the thresholds, *T*_*U*_ and *T*_*L*_, can be computed from the values of filter output *F*(*n*) by the least squares (LS) estimation method which minimizes the squared error between the observation *F*(*n*) and the threshold. According to the estimated threshold values, *T*_*U*_ and *T*_*L*_ are determined as 3.0 and −3.0 by averaging the filter output *F*(*n*) for over 10 sequential frames. The speech endpoint detection procedure is completed by mapping the result of the edge detection filter to an appropriate state transition model [16, 17]. However, the performance of the conventional frame energy based algorithm is degraded in noisy environments.  Figure 3(a) shows the speech signal at SNR of −5 dB in a car*-*noise environment while Figure 3(b) shows the beginning and ending points of clean speech obtained manually. As shown in Figure 3, the feature of the frame energy in noisy environment does not provide good results, as it is very unstable due to the large value of fluctuations. In Figure 3, it fails to detect the ending point of the speech and yields a slightly incorrect beginning point.

### 2.2. Static Harmonic Feature Based GMMs

This section outlines the procedure of extracting the harmonic features from noisy signals and describes how to use these features to discriminate between speech and nonspeech frames by using GMMs. The harmonic structures of speech and background noise are more distinguishable and more noise robust. Based on this argument, Fukuda et al. [18] extracted the harmonic structure based feature from the middle range of cepstral coefficients obtained from the discrete cosine transform (DCT) of the power spectral coefficients. The power spectrum of observed speech is first obtained by taking fast Fourier transform (FFT), which is then followed by taking logarithm to produce a log power spectrum *Y*_*k*_(*n*), where *n* and *k* are the frame number and frequency bin index, respectively. Then, a cepstrum *c*_*i*_(*n*) is obtained by applying DCT to the log power spectrum as

(3) $$\begin{array}{c} c_{i}\left(n\right)=a_{i}\overset{K}{\underset{k=1}{\sum }}Y_{k}\left(n\right)\cos\frac{\pi \left(2k-1\right)\left(i-1\right)}{2K},\;i=1,\ldots ,I,\\ a_{i}=\{\begin{array}{cc} \frac{1}{\sqrt{K}}, & \text{if}\;i=1\\ \sqrt{\frac{2}{K}}, & \text{otherwise}, \end{array} \end{array}$$

where *K* is the length of *Y*(*n*) and *i* is the cepstral index. The cepstral coefficients *c*_*i*_(*n*) with small and large indexes *i* are liftered out because they include long and short oscillations. Therefore, the following liftering process is applied to the cepstrum *c*_*i*_(*n*) as

(4) $$\begin{array}{c} {\hat{c}}_{i}\left(n\right)=\{\begin{array}{cc} \lambda c_{i}\left(n\right), & \text{if}\;\left(i<D_{L}\right),\left(i>D_{H}\right)\\ c_{i}\left(n\right), & \text{otherwise}, \end{array} \end{array}$$

where *λ* (=10^−3^) is a small constant and *D*_*L*_ and *D*_*H*_ are the lower and upper limit of cepstral indexes corresponding to the range of pitch frequencies in human voice. The liftered cepstrum ${\hat{c}}_{i}(n)$ is converted back to the log power spectrum *E*_*k*_(*n*) by taking an inverse discrete cosine transform (IDCT) and exponential transform to produce the linear power spectrum ${\hat{E}}_{k}(n)$. The coefficients ${\hat{E}}_{k}(n)$ are finally converted to mel*-*cepstrum ${\hat{q}}_{r}(n)$ by applying a mel*-*scale filter bank and DCT, where *r* is the bin number of the harmonic structure based mel*-*cepstral coefficients. This feature captures the envelope information of the local peaks in the frequency spectrum corresponding to the harmonic information in speech signals.

GMM based VAD is considered as a type of statistical model based VAD in which the feature vectors *y*(*n*) can be characterized by a mixture of Gaussian distributions. Hence, the decision rule in each frame is obtained by comparing the loglikelihood ratio Λ(*n*)  with decision threshold *η*:

(5) $$\begin{array}{c} \Lambda \left(n\right)=\log p\left(y\left(n\right)\;|\;H_{1}\right)-\log p\left(y\left(n\right)\;|\;H_{0}\right)\overset{H_{1}}{\underset{H_{0}}{≷}}\;\eta , \end{array}$$

where *p*(*y*(*n*)∣*H*_0_)  and  *p*(*y*(*n*)∣*H*_1_)  are the probability density functions of a speech absent frame and a speech present frame at* n* (*H*_0_: nonspeech GMM, *H*_1_: speech GMM), respectively.

This algorithm extracts and concatenates the two kinds of feature vectors (long*-*term temporal cepstra and harmonic structure information), which form the 26*-*dimensional feature vectors by using FFT, DCT, and IDCT in sequence [18]. However, since this algorithm extracts 26*-*dimensional features and transforms repeatedly between domains, it requires large and complex computations. Our proposed algorithm extracts computationally attractive and less burdening five*-*dimensional features that achieve effective and reliable VAD results, as verified by the CPU time and computational cost comparison experiment in Section 4.

## 3. The Proposed Line Fitting Model for Feature Set

**Figure 4:**
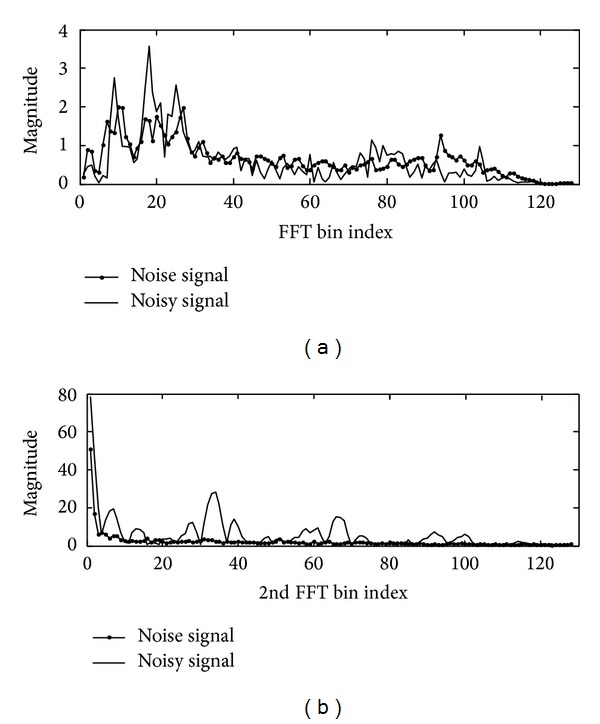
Difference in patterns of 1st FFT and 2nd FFT: (a) pattern of noisy speech signal and pattern of noise signal after 1st FFT and (b) pattern of noisy speech signal and pattern of noise signal after 2nd FFT.

The proposed feature extraction technique for VAD application is essentially based on DCFT and a line fitting procedure of the feature envelope, which is generally used for classification of noise sources. First, the DCFT output pattern (Section 3.1 and Figure 4) of a noisy speech (speech plus noise) signal and noise signal is obtained. Then, in order to describe the feature envelope using a small number of features, a feature envelope line fitting algorithm (Section 3.2) is developed. Finally, the distance between the input signal and estimated noise is utilized as a measure for segmentation (Section 3.3). In order to detect the activity of speech, the edge detection filter is applied to the distance measure over time.

### 3.1. Double*-*Combined Fourier Transform

Let signal *x*(*m*) be the noisy speech signal at time interval *m*. Signal *x*(*m*) is divided and overlapped into segments with 256-sample length and 128-sample length. Each segment is windowed, using a Hamming window, and then transformed via FFT. A frequency*-*domain representation *X*(*k*) of time*-*domain signal *x*(*m*) is obtained by

(6) $$\begin{array}{c} X\left(k\right)=\overset{M}{\underset{m=1}{\sum }}x\left(n\right)e^{-i(2\pi /M)km},\; \end{array}$$

where *k* and *M* are the frequency bin index and FFT point size, respectively. For the* k*th index, (6) becomes *X*(*k*)^1st^ and |*X*(*k*)^1st^| is the magnitude of the 1st FFT at index *k*. In order to proceed to the next FFT, it can be assumed that the magnitude of the 1st FFT is a time-domain signal, wherein the magnitude of the 1st FFT is considered as input to the 2nd FFT. Hence, 2nd FFT, *X*(*j*)^2nd^, is obtained by taking FFT of the magnitude of the 1st FFT, |*X*(*k*)^1st^|, as

(7) $$\begin{array}{c} X{\left(j\right)}^{2\text{nd}}=\overset{M}{\underset{k=1}{\sum }}\left|X{\left(k\right)}^{1\text{st}}\right|\;e^{-i(2\pi /M)jk}, \end{array}$$

where *j* is the DCFT (e.g., 2nd FFT) bin index and 256 FFT points are used for all FFT steps. This transform is wellsuited for analyzing harmonic components to compute the fundamental frequency of the signal. When a harmonic signal is observed, its Fourier transform has a series of peaks in its spectrum magnitude corresponding to the harmonics of the signal.  Figure 4(a) shows the magnitude of the 1st FFT of a noisy speech signal and that of a noise signal alone. It is difficult to distinguish between the two signals. Since there are harmonic signals that fluctuate periodically in the speech region, if the 2nd FFT is performed using the result of (a), the two signals can be better distinguished.

### 3.2. Line Fitting Feature Extraction

In this paper, the difference in the spectrum envelope pattern of the noise signal over the nonspeech region and that of the noisy speech signal over the speech region is exploited for information extraction. The resulting features of the proposed algorithm are effective when classifying the spectrum envelope patterns for various noise types and it is expected that the algorithm can be applied effectively to VAD. Five features are extracted from the resulting DCFT output by constructing two logarithmic curves as the best fit to the harmonic structure of the DCFT. The five extracted features include the mean index and the coefficients used to line-fit the envelope. It is expected that the proposed line fitting procedure separates speech cluster from noise effectively by capturing the essence of speech signal characteristics. Line fitting over the DCFT (e.g., 2nd FFT representing spectral dynamics) is essentially an envelope modulation of spectral dynamics conveying statistically significant amounts of information. As observed by many speech samples in noise, the first (lower frequency bins of DCFT) line fitting (e.g., first slope) captures low frequency energy bins whereas the second (higher frequency bin region of DCFT) line fitting (e.g., second slope) captures high frequency energy bins.

**Figure 5:**
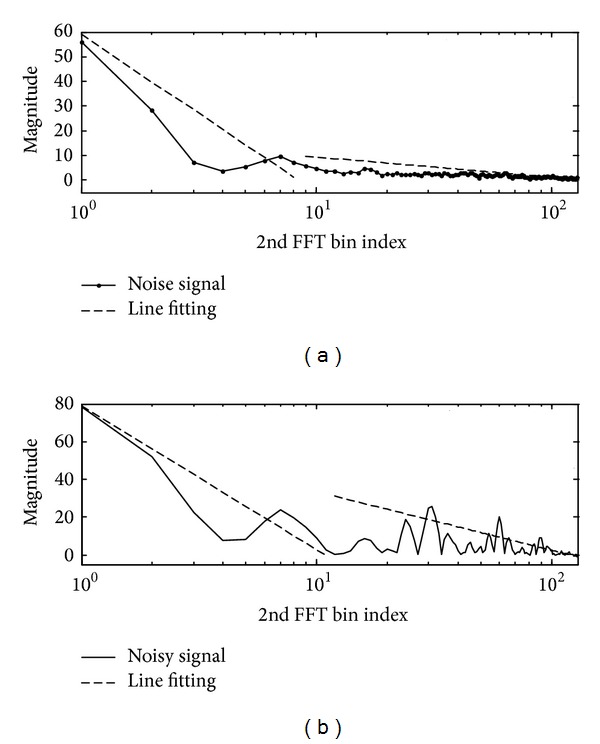
Result of fitting the line to spectrum magnitude obtained from the noise-only signal and the noisy signal of Figure 4(b): (a) line fitting to the magnitude of noise signal and (b) line fitting to the magnitude of noisy signal.

Figure 5 which is plotted on logarithmic frequency scale shows the lines that are fit by the magnitude of the DCFT obtained from the noise-only signal and the noisy signal of Figure 4(b), respectively. As shown in Figure 5, it is also observed in the DCFT bins that while the first slope of the noisy speech features overlaps quite well over the noise*-*only features, the second slope clearly shows a prominent difference as the noisy speech features display distinguishable harmonics above the floor made by noise-only features.

**Figure 6:**
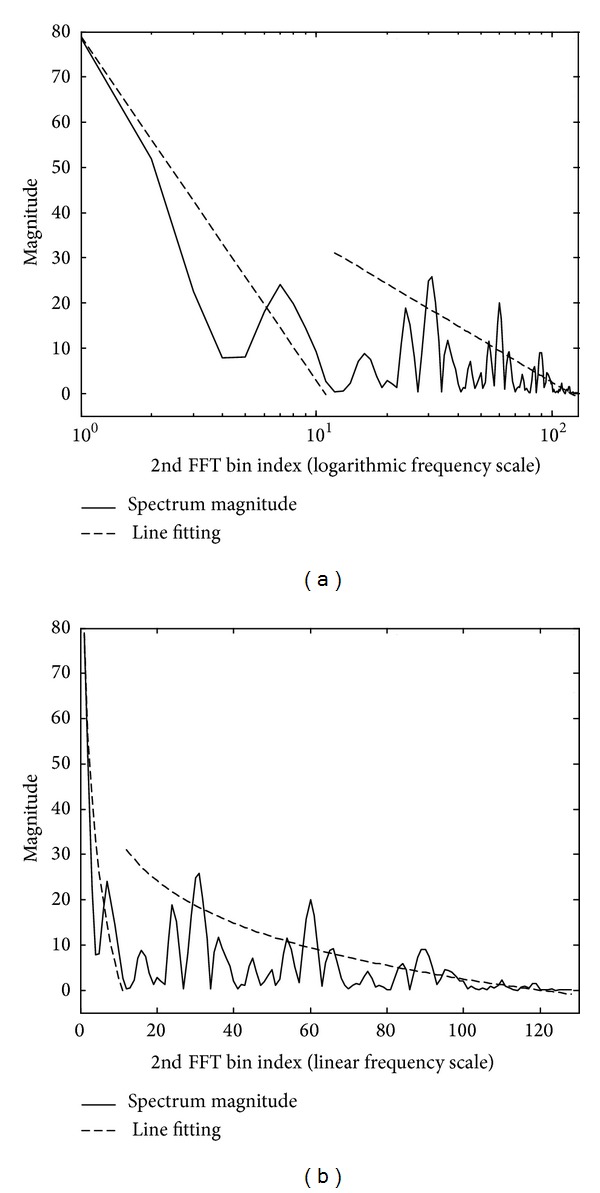
Result obtained from fitting the line to the spectrum magnitude: (a) line fitting to the magnitude in the linear frequency scale and (b) line fitting to the magnitude in dB in the logarithmic frequency scale.

Figure 6 shows the line fitting to the low frequency and high frequency slopes of the magnitude of the DCFT for the noisy speech signal.  Figure 6(a) was plotted on a logarithmic frequency scale while (b) was plotted on a linear frequency scale. As shown in Figure 6, the segmentation between the first line fitting and second line fitting is made by taking the mean index of the horizontal axis (e.g., DCFT bin index). It reflects the center of gravity of the DCFT energy bins, thus providing a solid dividing line between low frequency energy bins and high frequency energy bins. Rather than taking the full spectrum of the DCFT energy bins, the envelope modulation modeled by two slopes and mean index works quite well in successfully capturing the essence of critical information. As a result, the combined feature set (e.g., two line fittings in the form of slopes and mean index as separating point) can be used to effectively classify the speech over noise hypothesis.

The DCFT energy bins present a spectrum magnitude that can be described by determining the region having the concentration of spectral power and determining whether the spectrum is broadband or narrowband. This is accomplished by computing the first moment of the spectrum on a logarithmic frequency scale to give the mean frequency bin index. It provides the bin location at which the majority of the signal is contained. The mean frequency bin index of a spectrum is calculated as the sum of the product of the spectrum magnitude and the frequency bin index, divided by the total sum of spectrum magnitude. The mean frequency bin index is found by

(8) $$\begin{array}{c} F_{\text{mean}}=\frac{\sum_{j=1}^{N/2}\left|X{\left(j\right)}^{2\text{nd}}\right|}{\sum_{j=1}^{N/2}\left(\left|X{\left(j\right)}^{2\text{nd}}\right|/j\right)}=\frac{\sum_{j=1}^{N/2}|X{\left(j\right)}^{2\text{nd}}|j}{\sum_{j=1}^{N/2}\left|X{\left(j\right)}^{2\text{nd}}\right|}, \end{array}$$

where *F*_mean_ is the mean index, |*X*(*j*)^2nd^| is the magnitude of the DCFT at index* j*, and *N* is the size of the DCFT.

The spectrum is to be modeled by a pair of line fitting segments on a logarithmic frequency scale. The slopes of both the low and high indices are determined by fitting the line to the log response. One line fitting of the low index slope describes the envelope below the mean index, and the other line fitting of the high index slope describes the envelope above the mean index. Each line fitting model is given by

(9) $$\begin{array}{c} y\left(j\right)=a_{0}+a_{1}\text{lo}{\text{g}}_{2}\left(j\right), \end{array}$$

where *a*_0_ is a constant and *a*_1_ gives the slope. For the low index region, the mean*-*square error between the magnitude and the line fitting (first slope) to be minimized is given by

(10) $$\begin{array}{c} e=\overset{L}{\underset{j=1}{\sum }}\frac{1}{j}{\left[|X{\left(j\right)}^{2\text{nd}}|\;-y\left(j\right)\right]}^{\;2}, \end{array}$$

where *L* is the DCFT bin index just below the mean index and the factor 1/*j* is due to the logarithm frequency scale and degree of freedom used for the mean*-*square error.

The solution to the low index slope linear regression coefficients (*a*_*l*0_, *a*_*l*1_) is given by

(11) [al0al1]=[∑j=1L1j                      ∑j=1Llog2(j)j  ∑j=1Llog2(j)  j  ∑j=1L(log2(j))2j  ]−1 ×[∑j=1L|X(j)2nd|j∑j=1L|X(j)2nd|log2(j)j].

For the low index line fitting, index *j* of the summation goes from 1 to *L* and for the high index line fitting, index *j* of the summation goes from *L* + 1 to *N*/2. These modifications give, for the high index slope linear regression coefficients (*a*_*h*0_, *a*_*h*1_),

(12) [ah0ah1]=[∑j=L+1N/21j                        ∑j=L+1N/2log2(j)j∑j=L+1N/2log2(j)  j    ∑j=L+1N/2(log2(j))2j]−1 ×[∑j=L+1N/2|X(j)2nd|j∑j=L+1N/2|X(j)2nd|log2(j)j].

Finally, the mean index, the two coefficients (*a*_*l*0_, *a*_*l*1_) used for the envelope line fitting of the low indices, and the other two coefficients (*a*_*h*0_, *a*_*h*1_) used for the envelope line fitting of the high indices form the five*-*dimensional intermediate features.

### 3.3. Distance Measure for Segmentation

It is assumed that there are only noise signals in the first 10 frames (160 ms) and that the noise signal can be estimated by averaging. Then, a five*-*dimensional feature set representing the noise signal is obtained as a reference feature. At the next time point, another five*-*dimensional feature set is extracted frame by frame from the input signal. If the input signal is a noise signal, the input feature will be similar to the reference signal. On the contrary, if the speech signal is included in the current frame, the input feature will be different from the reference feature. With this motivation, a distance is proposed as a measure for segmentation. The distance, for example, at the *n*th frame can be represented as in

(13) $$\begin{array}{c} D\left(n\right)=\sqrt{\overset{P}{\underset{d=1}{\sum }}{\left(X_{\text{feat}}\left(d\right)-N_{\text{est}}\left(d\right)\right)}^{2}}, \end{array}$$

where *P* is the number of dimensions of the feature vector and *X*_feat_ and *N*_est_ represent the line fitting features of the input signal and estimated noise, respectively.

**Figure 7:**
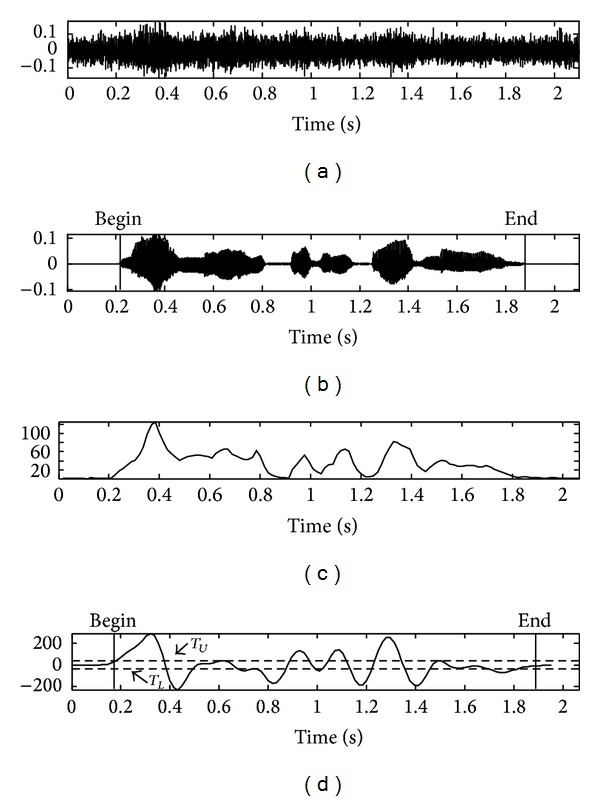
Results of the proposed algorithm in a car*-*noise environment: (a) noisy speech signal at SNR −5 dB, (b) clean speech signal, (c) the proposed feature, and (d) edge detection filter output based on the proposed feature.

**Figure 8:**
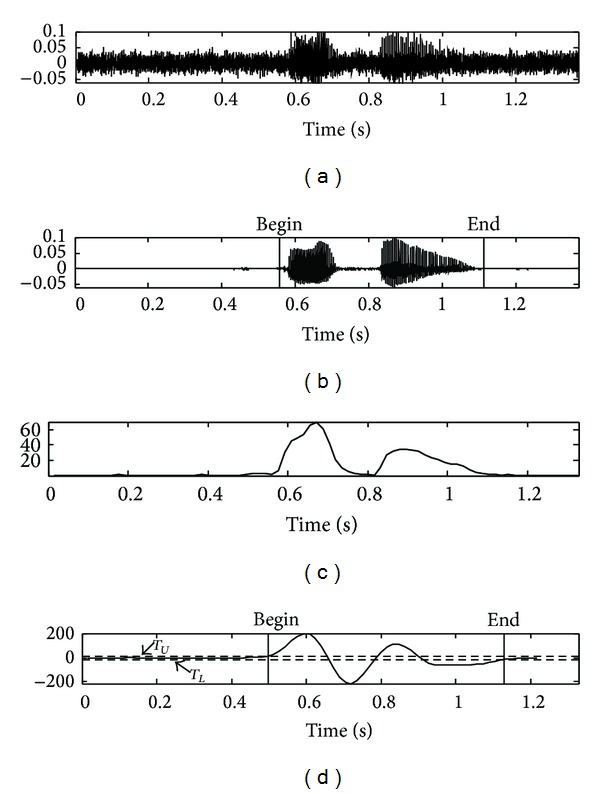
Results of the proposed algorithm in an exhibition hall*-*noise environment: (a) noisy speech signal at SNR −5 dB, (b) clean speech signal, (c) the proposed feature, and (d) edge detection filter output based on the proposed feature.

**Figure 9:**
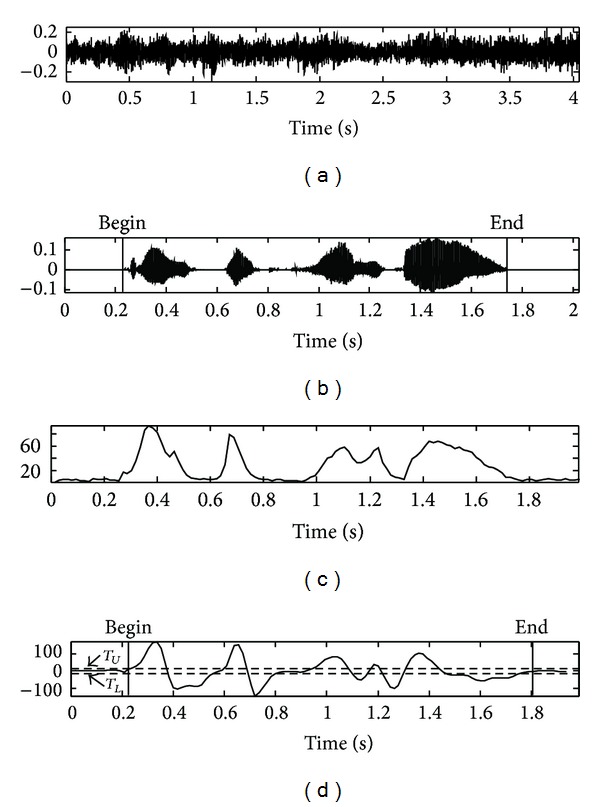
Results of the proposed algorithm in a babble*-*noise environment: (a) noisy speech signal at SNR −5 dB, (b) clean speech signal, (c) the proposed feature, and (d) edge detection filter output based on the proposed feature.

**Figure 10:**
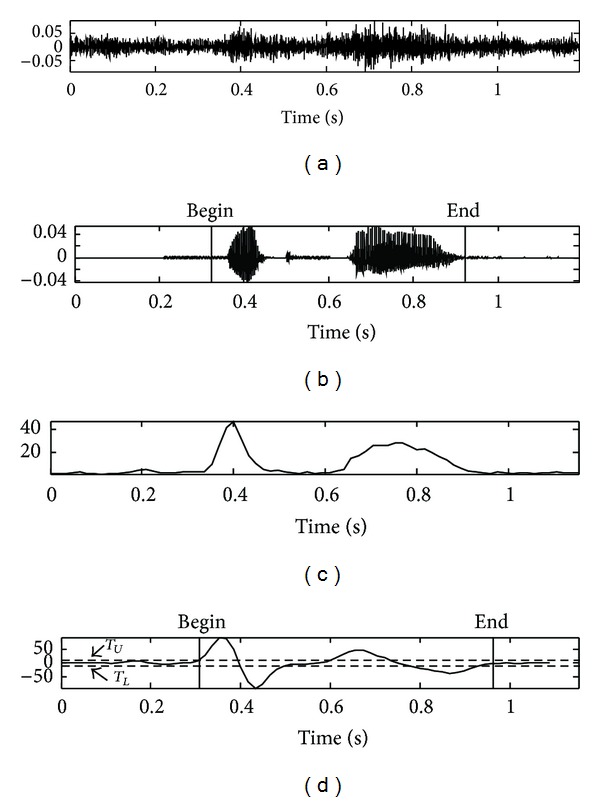
Results of the proposed algorithm in a restaurant*-*noise environment: (a) noisy speech signal at SNR −5 dB, (b) clean speech signal, (c) the proposed feature, and (d) edge detection filter output based on the proposed feature.

**Figure 11:**
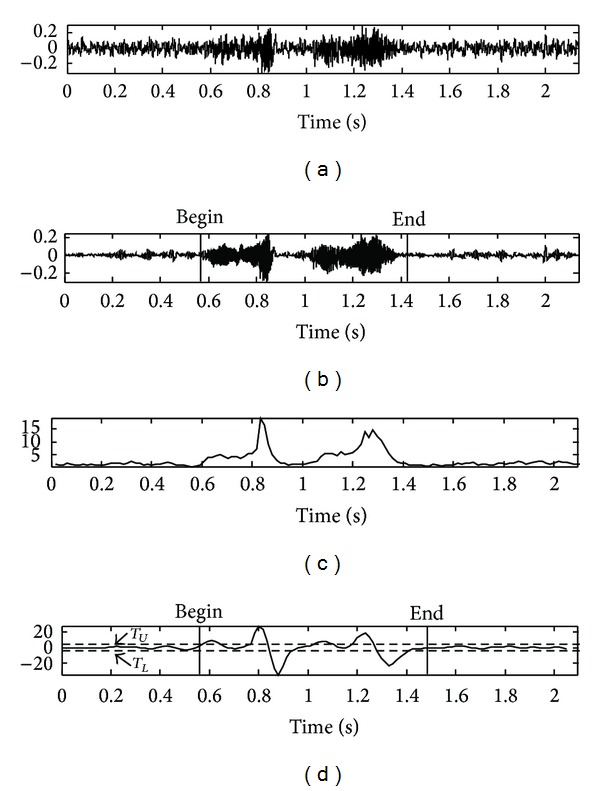
Results of the proposed algorithm in the environment of high speed driving on highway: (a) noisy speech signal at SNR −8 dB, (b) speech obtained manually by applying noise reduction [19] to (a) signal, (c) the proposed feature, and (d) edge detection filter output based on the proposed feature.

Figures 7, 8, 9, and 10 show the actual detection results in the case where the edge detection filter is applied to the distance measure of the speech data at −5 dB SNR in the car-, exhibition hall-, babble-, and restaurant-noise environment, respectively, as contained by the AURORA 2.0 database.  Figure 11 shows another result obtained from the experiment for the noisy speech collected under high speed driving condition on highway (−8 dB SNR on average), a case whose result of the proposed algorithm is applicable to the overall VAD performance comparison. This result indicates that the proposed VAD algorithm shows stable performance in real noise environment of high noise intensity as well.

## 4. VAD Performance Experiments

### 4.1. Experimental Setup

In this section, the performance of the proposed VAD algorithm is evaluated on the experimental data. Two well-known standard databases, AURORA 2.0 database and SITEC (Speech Information Technology & Industry Promotion Center) database, are employed for evaluations.

AURORA 2.0 database is a widely used training and testing standard database for speech recognition. Hence, it is appropriate as the database for evaluating VAD performance. It contains clean speech signal of English connected digits, and noisy speech signals have been recorded at different places representing both stationary noise (exhibition hall, car) and nonstationary noise (babble, restaurant) environments with varying SNRs including −5, 0, 5, 10, 15, and 20 dB. A total of 1,001 sentences are included in each SNR.

SITEC (Car03) is a database containing noisy speech collected from a microphone attached to the center of the sun*-*visor of a car at low speed (40–60 km/h) driving on city street and at high speed (70–90 km/h) driving on highway [20, 21]. The speech data were downsampled to 8 kHz for the experiments. 200 utterances, including a total of 400 Korean words collected at both low speed and high speed driving conditions, respectively, were used. In order to measure the intensity of the background noise for low speed driving and high speed driving, the SNR corresponding to each noise environment was obtained by means of a spectral subtraction procedure [19]. The obtained SNR shows −3 dB and −8 dB for low speed and high speed driving, respectively. The frame length for feature extraction is 256 samples (32 ms) while frame moving distance is 128 samples (16 ms).

### 4.2. Utterance Based Speech Segment Detection Test

First, we discuss effectiveness of the proposed VAD in terms of detecting speech segments correctly. In the experiment, a connected digit string is treated as an utterance in the AURORA 2.0 database. The beginning and ending points of the clean speech utterance were labeled manually based on the time unit. This manual detection information is used as a reference to determine the accuracy of those detected by the proposed algorithm. For the performance evaluation, the detection results of the conventional and proposed algorithm were compared to the ground truth reference. The following metrics are defined to evaluate the performance of the proposed algorithm.

- The probability of correctly detecting speech segments, *P*_*c*_, computed as the ratio of the number of correctly detected utterances to the total number of test utterances for each environment.
- The probability of falsely detecting speech segments, *P*_*f*_, computed as the ratio of the number of falsely detected utterances to the total number of test utterances for each environment.

Correct detection implies that there is no error or that the speech region is not damaged and includes those cases where the beginning point or ending point is equivalent or similar to the manual result. In other words, the detected segment (detected beginning point and ending point) is counted as correctly detected utterance in case it matches the manual result or when it is contained within the margins. The margin was set to about 0.08 seconds ahead of the manually detected beginning point and behind the ending point of the utterance. On the contrary, if either the detected beginning point is located behind the manually detected beginning point or the ending point is located ahead of the manually obtained result, false detection is admitted. In addition, if the detected beginning or ending point is beyond the margins, false detection is acknowledged.

The performance of the proposed algorithm was compared to that of the most prominent conventional algorithms (e.g., Sohn et al. [10], Ramírez et al. [14], Górriz et al. [15], Li et al. [17], and Fukuda et al. [18]). Note that the most recent conventional algorithms were selected among the edge detection based filtering procedures for performance comparison. Additionally, our algorithm was compared with the recent harmonic, long*-*term speech information and statistical based algorithms as well.

Table 1 shows the experimental results in terms of *P*_*c*_ values over various noise types and SNRs. In the experiments, high SNR includes speech data with clean 20, 15, and 10 dB signals, while the low SNR contains that with 5, 0, and −5 dB. As shown in Table 1, the average *P*_*c*_ using the proposed algorithm is higher compared to those using the conventional algorithms. This result demonstrates that the proposed feature set is robust against background noise, which essentially exploits the difference in the spectrum envelope pattern of the noise signal over the nonspeech region and that of the noisy speech signal over the speech region. The proposed algorithm shows average improvement of *P*_*c*_ by 5.9% over Sohn et al. [10], 0.9% over Ramírez et al. [14], 0.2% over Górriz et al. [15], 14.1% over Li et al. [17], and 2.7% over Fukuda et al. [18] for all representative realnoise environments considered. Li et al. [17] have the lowest performing results among the considered algorithms in the entirenoise environments. Furthermore, Li et al. [17] and Sohn et al. [10] suffered poor speech segment detection performance in low SNR conditions. While Ramírez et al. [14] and Górriz et al. [15] produced slightly better performance in babble and restaurant noise, they performed poorer in exhibition hall- and car-noise environments compared to the proposed algorithm. Detecting or recognizing only the target speaker's voice is difficult in nonstationary noise environment such as babble or restaurant which contains other people's voices as noise sources. In this situation, combining VAD with beamforming algorithm using a microphone array or blind source separation algorithm using nonnegative matrix factorization (NMF) seems promising.

### 4.3. Frame Based Speech and Nonspeech Discrimination Test

Second, the proposed VAD was evaluated in terms of the ability to discriminate between speech and nonspeech regions at different SNR levels. Again, the performance was measured using AURORA 2.0 database. The beginning and ending points of the clean speech utterance were obtained manually based on the frame unit. In order to evaluate the performance of the proposed VAD algorithm, experimental results were analyzed using two metrics which are known as nonspeech hit rate (HR0) and speech hit rate (HR1).

- HR0 is computed as the ratio of the number of correctly detected nonspeech frames to the number of real nonspeech frames.
- HR1 is computed as the ratio of the number of correctly detected speech frames to the number of real speech frames.

Since there are always trade*-*off relationships among these two metrics, the average of HR0 and HR1 is used as the metric for better performance comparison.  Table 2 compares the performance of the proposed VAD to conventional algorithms as mentioned in Section 4.2 for clean 20, 15, 10, 5, 0, and −5 dB. These results are averaged hit rates for the four types of noise considered in AURORA 2.0 database. In Table 2, we observed that the proposed algorithm achieved similar performance in detecting speech and nonspeech region when compared to Górriz et al. [15]. However, the proposed algorithm achieved better performance in detecting speech with 75.8% average value. It is demonstrated that the proposed algorithm produced an improvement in HR0 compared to the conventional ones.

**Figure 12:**
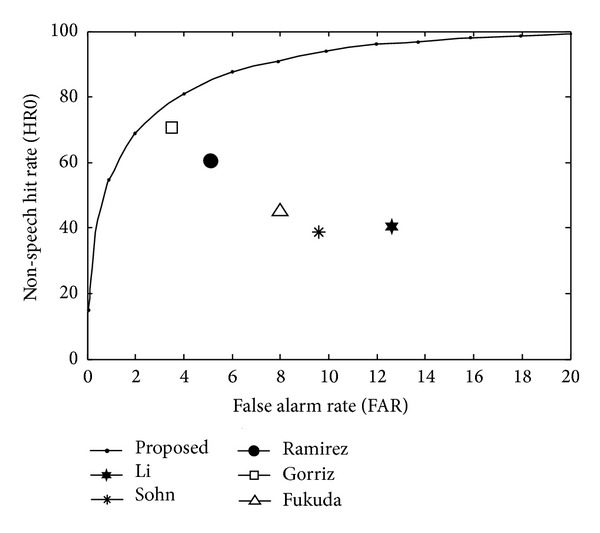
ROC curves: environment of high speed driving on highway (SNR −8 dB, 70–90 km/h).

An additional test was conducted to compare the speech and nonspeech detection performance in real driving environment by means of receiver operating characteristics (ROC). The ROC curves are used to completely describe the VAD error rate. The experiments were conducted using the SITEC databases Car03 of Korean speech recorded in driving car [20, 21]. Since the SITEC database used in the experiment does not contain clean speech, manual detection became a difficult task. Therefore, beginning point and ending point were obtained manually based on the frame unit after estimating the noisy speech with clean speech by using the spectral subtraction algorithm [19] for more accurate speech detection. This manual detection information is used as a reference to determine the accuracy of those detected by the proposed algorithm. The HR0 and the false alarm rate (FAR = 100*-*HR1) were determined in each noise condition.  Figure 12 shows the ROC curves of the proposed and conventional algorithms for the environment of high speed driving on highway (−8 dB SNR, 70–90 km/h). The results show improvements in detection accuracy over representative VAD algorithms. Thus, among all the VAD tested, our VAD algorithm resulted with the lowest FAR for a fixed HR0 and also the highest HR0 for a given FAR. As the experimental results show, the proposed DCFT and line fitting combined together become more robust features, especially in heavy noise under driving environment, compared to the conventional algorithm.

### 4.4. Computational Load and Robustness of the Proposed Feature Set

Another important performance measure is in terms of computational load. Additional advantage of the proposed algorithm is that the computational complexity created by the proposed algorithm is low. The computational complexity of the proposed algorithm was compared with that of its most comparably performing conventional algorithm (Górriz et al. [15]). Computation load aspect was separately evaluated in the two cases (proposed versus Górriz et al. [15]) in terms of CPU time and computational cost. To verify this, the CPU time was measured by performing on MATLAB with 2.9 GHz clock cycle and the computational cost based on the arithmetic operations with addition = 1, multiplication = 1, division = 5, and exponential = 10, for a noisy speech signal. Then the CPU time and computational cost of the proposed algorithm over the conventional algorithm were compared as shown in Table 3. As indicated by Table 3, the conventional algorithm required longer CPU time and higher computation cost compared to the proposed approach. It clearly shows that the proposed low*-*dimensional feature set is computationally less burdening and achieves more effective and reliable segmentation results.

In order to validate our proposition delineated in Section 3.2, we have conducted an additional set of experiments. In particular, an analysis of model clustering was conducted to investigate if the proposed feature extraction algorithm produces distinct segments. The proposed features were extracted from three sets of acoustic data (e.g., noise*-*only, noisy speech, and speech*-*only) and obtained three clusters from them.

Table 4 shows the experimental results, which indicate that the Euclidean distance between the noise*-*only and noisy speech cluster is similar to that between the noise*-*only and speech*-*only cluster, respectively, at various SNRs. As SNR decreases from −5 to 20 dB, however, the similarity between each cluster also tends to decrease but the overall error is confined to being small. It reinforces the fact that a set of five*-*dimensional features representing envelope modulation is quite effective for segmentation of noisy speech from that of noise*-*only regions.

## 5. Conclusions

This paper proposed a new VAD technique to improve automatic speech recognition innoisy environment. The proposed feature extraction technique, based on the DCFT and line fitting algorithm, was shown to be efficient and yet reliable. At the final step, the Euclidean distance was obtained as a measure for segmentation, and subsequently an edge detection filter was applied. Representative detection experiments conducted confirmed that the proposed algorithm is superior to the conventional algorithms by an average of 4.5% and 11.2%, in terms of correct detection probability and hit rate, respectively. In addition, the proposed algorithm confirms its superiority in terms of computational load and CPU time for processing compared to its most comparably performing conventional algorithm. Based on the analysis and results, the proposed low*-*complexity feature set is attractive and feasible for real*-*time implementation of VADs.

## Figures and Tables

**Table 1:** Comparison of the proposed algorithm to conventional algorithm in the utterance based speech segment detection test for various noise environments.

		Proposed	Sohn et al. [10]	Ramírez et al. [14]	Górriz et al. [15]	Li et al. [17]	Fukuda et al. [18]
Environments		*P*_*c*_ (%)	*P*_*c*_ (%)	*P*_*c*_ (%)	*P*_*c*_ (%)	*P*_*c*_ (%)	*P*_*c*_ (%)
Noise	SNR						
Babble	High	92.5	89.5	92.3	93.9	85.4	90.1
	Low	88.7	81.4	90.1	91.3	74.3	86.4
Restaurant	High	91.4	90.1	91.9	92.2	83.2	89.8
	Low	88.5	82.4	88.4	89.2	70.0	84.5
Exhibition hall	High	95.8	92.0	94.0	94.0	90.7	94.4
	Low	92.9	84.3	90.1	90.5	78.9	90.1
Car	High	95.9	91.5	94.5	94.5	87.5	94.1
	Low	92.5	86.4	90.5	91.0	77.4	88.8

Average		92.3	87.2	91.5	92.1	80.9	89.8

**Table 2:** Comparison of the proposed algorithm to conventional algorithm in the frame based speech and nonspeech discrimination test for SNRs from clean to −5 dB in terms of average HR0 and HR1.

	Proposed	Sohn et al. [10]	Ramírez et al. [14]	Górriz et al. [15]	Li et al. [17]	Fukuda et al. [18]
HR0	55.8	40.5	50.7	55.8	45.8	50.1
HR1	95.8	90.6	91.8	95.0	75.6	85.9

Average	75.8	65.6	71.2	75.4	60.7	68.0

**Table 3:** CPU time and computational cost comparison per single frame (=16 ms).

	Proposed	Górriz et al. [15]
CPU time	66.59 ms	81.45 ms
Computational cost	8990	9550

**Table 4:** The Euclidean distance between the three clusters.

SNR (dB)	−5	0	5	10	15	20
	Cluster 1					
	Noise-only	Noise-only	Noise-only	Noise-only	Noise-only	Noise-only

Clusters 2 and 3						
Noisy speech	8.5	12.7	15.9	19.1	22.8	25.5
Speech-only	5.4	9.9	12.9	17.2	21.3	24.3

Error	3.1	2.8	3.0	1.9	1.5	1.2
